# Refined stability of additive and quadratic functional equations in modular spaces

**DOI:** 10.1186/s13660-017-1422-z

**Published:** 2017-06-23

**Authors:** Hark-Mahn Kim, Hwan-Yong Shin

**Affiliations:** Department of Mathematics, Chungnam National University, 99 Daehangno, Yuseong-gu, Daejeon, 34134 Republic of Korea

**Keywords:** 39B52, 39B72, 47H09, modular functional, convex modular, Hyers-Ulam stability, Fatou property, $\Delta_{2}$-condition

## Abstract

The purpose of this paper is to obtain refined stability results and alternative stability results for additive and quadratic functional equations using direct method in modular spaces.

## Introduction

The theory of modulars on linear spaces and the related theory of modular linear spaces have been established by Nakano in 1950 [1]. Since then, these have been thoroughly developed by several mathematicians, for example, Amemiya [2], Koshi and Shimogaki [3], Yamamuro [4], Orlicz [5], Mazur [6], Musielak [7], Luxemburg [8], Turpin [9]. Up to now, the theory of modulars and modular spaces is widely applied in the study of interpolation theory [10, 11] and various Orlicz spaces [5].

First of all, we introduce to adopt the usual terminologies, notations, definitions and properties of the theory of modular spaces.

### Definition 1

Let X be a linear space over a field $\mathbb{K}$ ($\mathbb{R}$ or $\mathbb{C}$). We say that a generalized functional $\rho: X \rightarrow[0,\infty]$ is a modular if for any $x, y \in X$,

(M1): $\rho(x)=0$ if and only if $x=0$,
(M2): $\rho(\alpha x)=\rho(x)$ for all scalar *α* with $|\alpha|=1$,
(M3): $\rho(\alpha x + \beta y) \leq\rho(x)+\rho(y)$ for all scalar $\alpha, \beta\geq0$ with $\alpha+ \beta=1$.

If (M3) is replaced by

(M4): $\rho(\alpha x + \beta y) \leq\alpha\rho(x)+\beta\rho (y)$ for all scalar $\alpha, \beta\geq0$ with $\alpha+ \beta=1$, then the functional *ρ* is called a convex modular.

A modular *ρ* defines the following vector space:

$$\begin{aligned} X_{\rho}:= \bigl\{ x\in X : \rho(\lambda x) \rightarrow0 \text{ as } \lambda\rightarrow0 \bigr\} , \end{aligned}$$

and we say that $X_{\rho}$ is a modular space.

### Definition 2

Let $X_{\rho}$ be a modular space and let $\{ x_{n} \} $ be a sequence in $X_{\rho}$. Then:

1. $\{ x_{n} \} $ is *ρ*-convergent to a point $x \in X_{\rho}$ and write $x_{n} \xrightarrow{\rho} x $ if $\rho(x_{n}-x) \rightarrow0$ as $n \rightarrow\infty$.
2. $\{ x_{n} \} $ is called *ρ*-Cauchy if for any $\varepsilon>0$ one has $\rho(x_{n}-x_{m})<\varepsilon$ for sufficiently large $m, n \in\mathbb{N}$.
3. A subset $K \subseteq X_{\rho}$ is called *ρ*-complete if any *ρ*-Cauchy sequence is *ρ*-convergent to a point in *K*.

It is said that the modular *ρ* has the Fatou property if and only if $\rho(x) \leq\liminf_{n\rightarrow\infty}\rho(x_{n}) $ whenever the sequence $\{ x_{n} \}$ is *ρ*-convergent to *x* in modular space $X_{\rho}$.

### Proposition 1

*In modular spaces*,

1. *if* $x_{n} \xrightarrow{\rho} x$ *and* *a* *is a constant vector*, *then* $x_{n} +a \xrightarrow{\rho} x+a$, *and*
2. *if* $x_{n} \xrightarrow{\rho} x$ *and* $y_{n} \xrightarrow{\rho} y$, *then* $\alpha x_{n} + \beta y_{n} \xrightarrow{\rho} \alpha x + \beta y$, *where* $\alpha+ \beta\leq1$ *and* $\alpha, \beta\geq0$.

It is noticed that the convergence of a sequence $\{ x_{n}\}$ to *x* does not imply that $\{ cx_{n} \}$ converges to *cx* if *c* is chosen from the corresponding scalar field with $|c|>1$ in modular spaces. Thus, additional conditions on modular spaces were imposed by many mathematicians so that the multiples of convergent sequence $\{ x_{n}\}$ in the modular spaces converge naturally. A modular *ρ* is said to satisfy the △_2_-condition if there exists $k>0$ such that $\rho(2x)\leq k\rho(x)$ for all $x \in X_{\rho}$. Throughout this paper, we say that this constant *k* is a △_2_-constant related to $\Delta_{2}$-condition.

### Remark 1

Suppose that *ρ* is convex and satisfies △_2_-condition with △_2_-constant $k>0$. If $k<2$, then $\rho(x) \leq k \rho(\frac{x}{2})\leq\frac{k}{2}\rho (x) $, which implies $\rho=0$. Therefore, we must have the △_2_-constant $k \ge2 $ if *ρ* is convex modular.

The study of the stability of functional equations originated with Ulam [12], who raised the stability problem of group homomorphisms. Hyers [13] gave the first affirmative answer to Ulam’s question in the case of a Cauchy functional equation in Banach spaces. In honor of the Hyers answer to the question of Ulam, the stability of functional equations may be called Hyers-Ulam stability. Hyers’ approach to proving Ulam’s problem, which is often called the direct method [13], has been extensively used for studying the stability of various functional equations [14, 15]. Additionally, there are also other methods proving the Hyers-Ulam stability of some functional equations [16], for example, the method using the property of shadowing [17], the method of invariant means [18], the method based on sandwich theorems [19]. The most popular technique of proving the stability of functional equations except for direct method is the fixed point method [16, 20–23].

On the other hand, many authors have investigated the stability using fixed point theorem of quasicontraction mappings in modular spaces without $\Delta_{2}$-condition, which has been introduced by Khamsi [24]. Recently, the stability results of additive functional equations in modular spaces equipped with the Fatou property and $\Delta _{2}$-condition were investigated by Sadeghi [25] who used Khamsi’s fixed point theorem. Also the stability of quadratic functional equations in modular spaces satisfying the Fatou property without using the $\Delta_{2}$-condition was proved by Wongkum, Chaipunya and Kumam [26].

In this paper, by using the direct method, we present stability results and alternative stability results of additive functional equations and of quadratic functional equations which are refined versions of Sadeghi [25], and Wongkum, Chaipunya and Kumam [26].

## Stability of additive functional equations in modular spaces

Throughout this paper, we assume that *V* is a linear space and $X_{\rho}$ is a *ρ*-complete convex modular space. We present a main theorem, which concerns Hyers-Ulam stability of an additive functional equation in modular spaces without using the Fatou property.

### Theorem 1

*Suppose* $X_{\rho}$ *satisfies the* $\Delta_{2}$-*condition*. *If there exists a function* $\varphi:V^{2} \to[0,\infty)$ *for which a mapping* $f:V\to X_{\rho}$ *satisfies*

1 $$ \begin{aligned}[t] & \rho\bigl(f(x+y)-f(x)-f(y)\bigr) \leq \phi(x,y), \\ & \lim_{n\rightarrow\infty} k^{n}\phi {\biggl(} \frac{x}{2^{n}}, \frac {y}{2^{n}} {\biggr)}=0 \quad \textit{and} \quad \sum _{i=1}^{\infty} {\biggl(}\frac{k^{2}}{2} { \biggr)}^{i} \phi {\biggl(}\frac{x}{2^{i}},\frac{x}{2^{i}} { \biggr)}< \infty \end{aligned} $$

*for all* $x, y \in V$, *then there exists a unique additive mapping* $A:V \to X_{\rho}$, *defined as* $A(x)=\lim_{n\to\infty}2^{n}f(\frac{x}{2^{n}}) $ *and*

2 $$ \rho\bigl( f(x)-A(x) \bigr) \leq\frac{1}{2} \sum _{i=1}^{\infty} {\biggl(}\frac {k^{2}}{2} { \biggr)}^{i}\phi {\biggl(}\frac{x}{2^{i}},\frac{x}{2^{i}} {\biggr)} $$

*for all* $x\in V$.

### Proof

By letting $x, y$ by $\frac{x}{2}$ in (1), respectively, we get

$$ \phi {\biggl(} f(x)-2f {\biggl(} \frac{x}{2} {\biggr)} {\biggr)} \leq \phi {\biggl(} \frac{x}{2},\frac{x}{2} {\biggr)} $$

for all $x\in V$, and then it follows from the $\Delta_{2}$-condition and the convexity of the modular *ρ* that

$$ \begin{aligned} \rho {\biggl(} f(x) - 2^{n}f {\biggl(} \frac{x}{2^{n}} {\biggr)} { \biggr)} &= \rho {\Biggl(} \sum_{i=1}^{n} \frac{1}{2^{i}} {\biggl(} 2^{2i-1}f {\biggl(} \frac {x}{2^{i-1}} { \biggr)} -2^{2i}f {\biggl(} \frac{x}{2^{i}} {\biggr)} {\biggr)} { \Biggr) } \\ &\leq \frac{1}{k}\sum_{i=1}^{n} { \biggl(}\frac{k^{2}}{2} {\biggr)}^{i}\phi {\biggl(} \frac{x}{2^{i}}, \frac{x}{2^{i}} {\biggr)} \end{aligned}$$

for all $x \in V$. So, for all $n,m \in\mathbb{N}$ with $n \geq m$, we have

$$ \begin{aligned} \rho {\biggl(} 2^{n}f {\biggl(} \frac{x}{2^{n}} { \biggr)}-2^{m}f {\biggl(} \frac {x}{2^{m}} {\biggr)} {\biggr)} & \leq k^{m}\rho {\biggl(} 2^{n-m}f {\biggl(} \frac{x}{2^{n}} { \biggr)} - f {\biggl(} \frac{x}{2^{m}} {\biggr)} {\biggr)} \\ & \leq k^{m-1} \sum_{i=1}^{n-m} { \biggl(}\frac{k^{2}}{2} {\biggr)}^{i}\phi {\biggl(} \frac{x}{2^{m+i}},\frac{x}{2^{m+i}} {\biggr)} \\ & = \frac{1}{k} {\biggl(} \frac{2}{k} {\biggr)}^{m} \sum_{i=m+1}^{n} {\biggl(}\frac{k^{2}}{2} {\biggr)}^{i}\phi {\biggl(} \frac{x}{2^{i}}, \frac{x}{2^{i}} {\biggr)} \end{aligned}$$

for all $x \in V$. Since the right-hand side of the above inequality tends to zero as *m* goes to infinity, the sequence $\{ 2^{n}f(\frac {x}{2^{n}}) \}$ is a *ρ*-Cauchy sequence in $X_{\rho}$ and so the sequence $\{ 2^{n}f {(} \frac{x}{2^{n}} {)} \} $ is a *ρ*-convergent sequence on $X_{\rho}$. Thus, we may define a mapping $A :V \to X_{\rho}$ as

$$ A (x):= \text{$\rho$-}\lim_{n\to\infty} 2^{n}f {\biggl(} \frac{x}{2^{n}} {\biggr)}, \quad \textit{i.e.}, \lim _{ {n\to\infty} } \rho {\biggl(} 2^{n}f {\biggl(} \frac{x}{2^{n}} {\biggr)} - A (x) {\biggr)} =0 \quad (x \in V). $$

According to the $\Delta_{2}$-condition without using the Fatou property, we obtain the following inequality:

$$ \begin{aligned} \rho\bigl(f(x) - A(x)\bigr) &\le \frac{1}{2} \rho {\biggl(} 2f(x) - 2^{n+1} f {\biggl(} \frac{x}{2^{n}} {\biggr)} {\biggr)} + \frac{1}{2}\rho {\biggl(} 2^{n+1} f {\biggl(} \frac{x}{2^{n}} { \biggr)}- 2 A(x) {\biggr)} \\ &\leq \frac{k}{2} \rho {\biggl(}f(x)-2^{n} f {\biggl(} \frac{x}{2^{n}} {\biggr)} {\biggr)} + \frac{k}{2}\rho {\biggl(} 2^{n} f {\biggl(} \frac{x}{2^{n}} {\biggr)}- A(x) {\biggr)} \\ &\leq \frac{1}{2} \sum_{i=1}^{n} {\biggl(}\frac{k^{2}}{2} {\biggr)}^{i} \phi {\biggl(} \frac{x}{2^{i}} ,\frac{x}{2^{i}} {\biggr)} +\frac{k}{2}\rho {\biggl(} 2^{n} f {\biggl(}\frac{x}{2^{n}} {\biggr)}-A(x) {\biggr)} \end{aligned}$$

for all $x \in V$. Taking $n \rightarrow\infty$, we conclude that the estimation (2) of *f* by *A* holds for all $x \in V$.

Now, we claim that the mapping *A* is additive. Setting $(x, y) := (2^{-n}x, 2^{-n}y)$ in (1) and using the $\Delta_{2}$-condition, we see that

$$ \rho {\biggl(} 2^{n}f {\biggl(} \frac{x+y}{2^{n}} {\biggr)} - 2^{n}f {\biggl(} \frac {x}{2^{n}} {\biggr)} - 2^{n}f { \biggl(} \frac{y}{2^{n}} {\biggr)} {\biggr)} \leq k^{n}\phi {\biggl(} \frac{x}{2^{n}},\frac{y}{2^{n}} {\biggr)} $$

for all $x ,y \in V$. Thus, it follows from the $\Delta_{2}$-condition and $\rho(\alpha x) \leq\alpha\rho(x)$ ($0\leq\alpha\leq1$, $x \in V$) that

$$\begin{aligned} \rho {\bigl(} A(x+y)-A(x)-A(y) {\bigr)} \leq{}& \frac{1}{4}\rho {\biggl(}4 {\biggl(} A(x+y)-2^{n}f {\biggl(} \frac {x+y}{2^{n}} {\biggr)} { \biggr)} {\biggr)} \\ &+ \frac{1}{4}\rho {\biggl(}4 {\biggl(} A(x)-2^{n}f { \biggl(} \frac {x}{2^{n}} {\biggr)} {\biggr)} {\biggr)} \\ & + \frac{1}{4}\rho {\biggl(}4 {\biggl(} A(y)-2^{n}f { \biggl(} \frac {y}{2^{n}} {\biggr)} {\biggr)} {\biggr)} \\ & +\frac{1}{4}\rho {\biggl(} 4 {\biggl(} 2^{n}f {\biggl(} \frac{x+y}{2^{n}} {\biggr)} - 2^{n}f {\biggl(} \frac{x}{2^{n}} { \biggr)} - 2^{n}f {\biggl(} \frac {y}{2^{n}} {\biggr)} {\biggr)} { \biggr)} \\ \leq{}& \frac{k^{2}}{4}\rho {\biggl(} A(x+y)-2^{n}f {\biggl(} \frac {x+y}{2^{n}} {\biggr)} {\biggr)} \\ & + \frac{k^{2}}{4}\rho {\biggl(} A(x)-2^{n}f {\biggl(} \frac{x}{2^{n}} {\biggr)} {\biggr)} \\ & + \frac{k^{2}}{4}\rho {\biggl(} A(y)-2^{n}f {\biggl(} \frac{y}{2^{n}} {\biggr)} {\biggr)} \\ & +\frac{k^{2}}{4}\rho {\biggl(} 2^{n}f {\biggl(} \frac{x+y}{2^{n}} {\biggr)} - 2^{n}f {\biggl(} \frac{x}{2^{n}} { \biggr)} - 2^{n}f {\biggl(} \frac{y}{2^{n}} {\biggr)} {\biggr)} \end{aligned}$$

for all $x , y\in V$ and all positive integers *n*. Taking the limit as $n\rightarrow\infty$, one sees that *A* is additive.

To show the uniqueness of *A*, we assume that there exists an additive mapping $A' : V \rightarrow X_{\rho}$ which satisfies the inequality

$$ \rho\bigl(f(x)-A'(x)\bigr) \leq\frac{1}{2}\sum _{i=1}^{\infty} {\biggl(}\frac {k^{2}}{2} { \biggr)}^{i} \phi {\biggl(}\frac{x}{2^{i}}, \frac{x}{2^{i}} { \biggr)} $$

for all $x \in V$. Then, since *A* and $A'$ are additive mappings, we see from the equality $A(2^{-n}x)=2^{-n}A(x)$ and $A'(2^{-n}x)=2^{-n}A'(x)$ that

$$ \begin{aligned} \rho\bigl(A(x)-A'(x)\bigr) \leq{}& \frac{1}{2}\rho {\biggl(} 2^{n+1}A {\biggl(} \frac {x}{2^{n}} {\biggr)}-2^{n+1}f { \biggl(} \frac{x}{2^{n}} {\biggr)} {\biggr)} \\ & + \frac{1}{2}\rho {\biggl(} 2^{n+1}f {\biggl(} \frac{x}{2^{n}} {\biggr)} - 2^{n+1}A' {\biggl(} \frac{x}{2^{n}} {\biggr)} {\biggr)} \\ \leq{}&\frac{k^{n+1}}{2}\rho {\biggl(} A {\biggl(} \frac{x}{2^{n}} { \biggr)}-f {\biggl(} \frac{x}{2^{n}} {\biggr)} {\biggr)} + \frac{k^{n+1}}{2} \rho {\biggl(}f {\biggl(} \frac{x}{2^{n}} {\biggr)} - A' {\biggl(} \frac{x}{2^{n}} {\biggr)} {\biggr)} \\ \leq{}& \frac{k^{n+1}}{2}\sum_{i=1}^{\infty} { \biggl(}\frac{k^{2}}{2} {\biggr)}^{i} \phi {\biggl(} \frac{x}{2^{n+i}}, \frac{x}{2^{n+i}} {\biggr)} \\ \leq{}& {\biggl(}\frac{2}{k} {\biggr)}^{n-1}\sum _{i=n+1}^{\infty} {\biggl(}\frac{k^{2}}{2} { \biggr)}^{i} \phi {\biggl(}\frac{x}{2^{i}}, \frac {x}{2^{i}} { \biggr)} \end{aligned}$$

for all $x \in V$ and all positive integers *n*. Hence *A* is a unique additive mapping near *f* satisfying the approximation (2) in the modular space $X_{\rho}$. This completes the proof. □

### Corollary 1

*Suppose* *V* *is a normed space with norm* $\| \cdot\|$ *and* $X_{\rho}$ *satisfies* $\Delta_{2}$-*condition*. *For given real numbers* $\theta>0$ *and* $p>\log_{2}\frac{k^{2}}{2}$, *if* $f:V \rightarrow X_{\rho}$ *is a mapping such that*

$$ \rho\bigl(f(x+y)-f(x)-f(y)\bigr)\leq\theta\bigl(\| x \|^{p}+\| y \|^{p}\bigr) $$

*for all* $x, y \in V$, *then there exists a unique additive mapping* $A:V \rightarrow X_{\rho}$ *such that*

$$ \rho\bigl(f(x)-A(x)\bigr) \leq\frac{k^{2}\theta}{2^{p+1}-k^{2}}\|x\|^{p} $$

*for all* $x \in V$.

Next, we are going to prove an alternative stability theorem of additive functional equations in modular spaces without using the $\Delta_{2}$-condition.

### Theorem 2

*Let* $X_{\rho}$ *satisfy the Fatou property*. *Suppose that a mapping* $f : V \rightarrow X_{\rho} $ *satisfies*

3 $$ \rho\bigl(f(x+y)-f(x)-f(y)\bigr)\leq\phi(x,y) $$

*and* $\phi:V \times V \rightarrow[0, \infty) $ *is a mapping such that*

$$ \lim_{n\rightarrow\infty} \frac{\phi(2^{n}x,2^{n}y)}{2^{n}}=0, \qquad\sum _{i=0}^{\infty}\frac{\phi(2^{i}x,2^{i}x)}{2^{i}}< \infty $$

*for all* $x, y \in V$. *Then there exists a unique additive mapping* $A: V \rightarrow X_{\rho}$ *such that*

4 $$ \rho\bigl(f(x)-A(x)\bigr) \leq\frac{1}{2}\sum _{i=0}^{\infty}\frac{\phi(2^{i}x, 2^{i}x)}{2^{i}} $$

*for all* $x \in V$.

### Proof

We let $y=x$ in (3) and have

$$ \rho\bigl(f(2x)-2f(x)\bigr)\leq\phi(x,x), $$

so we observe without using the △_2_-condition that

$$ \begin{aligned} \rho {\biggl(}\frac{f(2^{n}x)}{2^{n}}-f(x) {\biggr)} ={}& \rho {\Biggl(} \sum _{i=0}^{n-1}\frac{1}{2^{i+1}}\bigl(2f \bigl(2^{i}x\bigr)-f\bigl(2^{i+1}x\bigr)\bigr) {\Biggr)} \\ \leq{}& \sum_{i=0}^{n-1}\frac{1}{2^{i+1}} \rho {\bigl(} \bigl(2f\bigl(2^{i}x\bigr)-f\bigl(2^{i+1}x\bigr) \bigr) {\bigr)} \\ \leq{}& \frac{1}{2}\sum_{i=0}^{n-1} \frac{1}{2^{i}}\phi\bigl( 2^{i} x, 2^{i} x\bigr) \end{aligned} $$

for all $x \in V$ and all positive integers $n>1$. This yields

$$ \begin{aligned} \rho {\biggl(} \frac{f(2^{n}x)}{2^{n}}-\frac{f(2^{m}x)}{2^{m}} {\biggr)} & \leq \frac{1}{2^{m}}\rho {\biggl(} \frac{ f(2^{n-m}\cdot2^{m}x)}{2^{n-m}}- f\bigl(2^{m}x\bigr) {\biggr)} \\ &\leq \frac{1}{2^{m}}\sum_{i=0}^{n-m-1} \frac{1}{2^{i+1}}\phi\bigl( 2^{i}\cdot2^{m}x, 2^{i} \cdot2^{m}x\bigr) \\ & = \frac{1}{2}\sum_{i=m}^{n-1} \frac{1}{2^{i}}\phi\bigl( 2^{i} x, 2^{i} x\bigr) \end{aligned}$$

for all $x \in V$ and all $n, m \in\mathbb{N}$ with $n>m$. Thus, we see that the sequence $\{ \frac{f(2^{n}x)}{2^{n}}\} $ is a *ρ*-Cauchy sequence on $X_{\rho}$. Since $X_{\rho}$ is *ρ*-complete, there exists a *ρ*-limit function $A:V \rightarrow X_{\rho}$ defined by

$$ \text{$\rho$-}\lim_{n\rightarrow\infty} \frac {f(2^{n}x)}{2^{n}}:=A(x),\quad \textit{i.e.}, \lim_{n \rightarrow\infty}\rho {\biggl(}\frac{f(2^{n}x)}{2^{n}}-A(x) { \biggr)}=0 $$

for all $x \in V$. Then, it follows from the Fatou property that the inequality

$$ \rho {\bigl(}A(x)-f(x) {\bigr)}\leq\liminf_{n\rightarrow\infty}\rho {\biggl(} \frac{f(2^{n}x)}{2^{n}}-f(x) {\biggr)} \leq\frac{1}{2}\sum _{i=0}^{\infty} \frac{1}{2^{i}}\phi\bigl( 2^{i} x, 2^{i} x\bigr) $$

holds for all $x \in V$. Now, we claim that *A* satisfies the additive functional equation. Note that

$$ \rho {\biggl(} \frac{f(2^{n}(x+y))}{2^{n}}-\frac{f(2^{n}x)}{2^{n}}-\frac {f(2^{n}y)}{2^{n}} { \biggr)} \leq\frac{1}{2^{n}}\phi\bigl(2^{n}x,2^{n}y\bigr) $$

for all $x,y \in V $ and all $n \in\mathbb{N}$. Thus, we observe by convexity of *ρ* that

$$\begin{aligned} \rho {\biggl(}\frac{1}{4}A(x+y)-\frac{1}{4}A(x)- \frac{1}{4}A(y) {\biggr)} \leq{}& \frac{1}{4}\rho {\biggl(} A(x+y)- \frac{f(2^{n}(x+y))}{2^{n}} {\biggr)} \\ & + \frac{1}{4}\rho {\biggl(} A(x)- \frac{f(2^{n}x)}{2^{n}} {\biggr)} \\ & +\frac{1}{4}\rho {\biggl(} A(y)- \frac{f(2^{n}y)}{2^{n}} {\biggr)} \\ & +\frac{1}{4}\rho {\biggl(} \frac{f(2^{n}(x+y))}{2^{n}}-\frac {f(2^{n}x)}{2^{n}}- \frac{f(2^{n}y)}{2^{n}} {\biggr)} \end{aligned}$$

holds for all $x , y \in V$, and then taking $n \rightarrow\infty$, one obtains $\rho(\frac{1}{4} (A(x+y)-A(x)-A(y)))=0$. This implies that *A* is additive.

To show the uniqueness of *A*, we assume that there exists another additive mapping $A' : V \rightarrow X_{\rho}$ near *f* satisfying the approximation (4). Since *A* and $A'$ are additive mappings, we see from the equality $A(2^{n}x)=2^{n}A(x)$ and $A'(2^{n}x)=2^{n}A'(x)$ that

$$\begin{aligned} \rho {\biggl(}\frac{1}{2} A(x)- \frac{1}{2}A'(x) { \biggr)} &\leq \frac {1}{2}\rho {\biggl(}\frac{A(2^{n}x)}{2^{n}}- \frac{f(2^{n}x)}{2^{n}} {\biggr)} + \frac{1}{2}\rho {\biggl(}\frac{f(2^{n}x)}{2^{n}}- \frac{A'(2^{n}x)}{2^{n}} {\biggr)} \\ & \leq \frac{1}{2^{n+1}}\rho {\bigl(} A\bigl(2^{n}x\bigr)-f \bigl(2^{n}x\bigr) {\bigr)} + \frac {1}{2^{n+1}}\rho {\bigl(} f \bigl(2^{n}x\bigr)-A'\bigl(2^{n}x\bigr) {\bigr)} \\ & \leq \frac{1}{2^{n}}\sum_{i=0}^{\infty} \frac{1}{2^{i+1}}\phi\bigl( 2^{i}\cdot2^{n}x, 2^{i} \cdot2^{n}x\bigr) \\ & = \sum_{i=n}^{\infty} \frac{1}{2^{i+1}}\phi \bigl( 2^{i}x, 2^{i}x\bigr) \end{aligned}$$

for all $x \in V$. Taking $n \rightarrow\infty$, we find that $A = A'$. Hence *A* is a unique additive mapping near *f* satisfying the approximation (4). □

### Remark 2

In particular, if $X_{\rho}$ is a Banach space with norm *ρ*, then $\rho(2x)=2\rho(x)$, $k=2$, and so Theorem 2 is equivalent to the result of Gǎvruta [14] in this case.

The following corollary, which does not use △_2_-condition of *ρ*, is a refined version of Sadeghi’s stability result (Theorem 2.1 in [25]) in modular space $X_{\rho}$.

### Corollary 2

*Let* $X_{\rho}$ *satisfy the Fatou property*. *Suppose that a mapping* $f : V \rightarrow X_{\rho} $ *satisfies*

$$ \rho\bigl(f(x+y)-f(x)-f(y)\bigr)\leq\phi(x,y) $$

*and* $\phi:V \times V \rightarrow[0, \infty) $ *is a mapping such that*

$$ \lim_{n\rightarrow\infty} \frac{\phi(2^{n}x,2^{n}y)}{2^{n}}=0, \qquad \phi(2x,2x)\leq2L \phi(x,x) $$

*for all* $x, y \in V$. *Then there exists a unique additive mapping* $A: V \rightarrow X_{\rho}$ *such that*

$$ \rho\bigl(f(x)-A(x)\bigr) \leq\frac{1}{2(1-L)}\phi(x,x) $$

*for all* $x \in V$.

### Corollary 3

*Let V be a normed space with norm* $\| \cdot\| $ *and* $X_{\rho}$ *satisfy the Fatou property*. *For given real numbers* $\theta, \varepsilon >0$ *and* $p \in(-\infty, 1)$, *if* $f:V \rightarrow X_{\rho}$ *is a mapping such that*

$$ \rho\bigl(f(x+y)-f(x)-f(y)\bigr)\leq\theta\bigl(\| x \|^{p}+\| y \|^{p}\bigr)+\varepsilon $$

*for all* $x, y \in V$, *then there exists a unique additive mapping* $A: V \rightarrow X_{\rho}$ *such that*

$$ \rho\bigl(f(x)-A(x)\bigr) \leq\frac{2\theta}{2-2^{p}}\|x\|^{p}+\varepsilon $$

*for all* $x \in V$, *where* $x \neq0$ *if* $p < 0$.

## Stability of quadratic functional equations in modular spaces

In this section, we investigate refined stability results of the original quadratic functional equation in modular space $X_{\rho}$. We present the Hyers-Ulam stability of a quadratic functional equation in modular spaces without using the Fatou property.

### Theorem 3

*Suppose* $X_{\rho}$ *satisfies the* $\Delta_{2}$-*condition*. *If there exists a function* $\varphi:V^{2} \to[0,\infty)$ *for which a mapping* $f:V \to X_{\rho}$ *satisfies*

5 $$ \begin{aligned}[t] & \rho\bigl(f(x+y)+f(x-y)-2f(x)-2f(y)\bigr) \leq \phi(x,y), \\ & \lim_{n\rightarrow\infty} k^{2n}\phi {\biggl(} \frac{x}{2^{n}},\frac {y}{2^{n}} {\biggr)}=0 \quad\textit{and} \quad \sum _{i=1}^{\infty} {\biggl(}\frac{k^{3}}{2} { \biggr)}^{i} \phi {\biggl(}\frac{x}{2^{i}},\frac{x}{2^{i}} { \biggr)}< \infty \end{aligned}$$

*for all* $x, y \in V$, *then there exists a unique quadratic mapping* $B:V \to X_{\rho}$, *defined as* $B(x)=\lim_{n\to\infty}4^{n}f(\frac{x}{2^{n}}) $ *and*

6 $$ \rho\bigl(f(x)-B(x)\bigr) \leq\frac{1}{2k}\sum _{i=1}^{\infty} {\biggl(}\frac {k^{3}}{2} { \biggr)}^{i}\phi {\biggl(} \frac{x}{2^{i}}, \frac{x}{2^{i}} { \biggr)} $$

*for all* $x\in V$.

### Proof

First, we observe that $f(0) = 0$ because of $\phi(0,0)=0$ by the convergence of $\sum_{i=1}^{\infty} {(}\frac{k^{3}}{2} {)}^{i}\phi(0,0)<\infty$. We take $y=x $ in (5) to have

$$ \rho\bigl(f(2x)-4f(x)\bigr)\leq\phi(x,x) $$

for all $x \in V$. By the $\Delta_{2}$-condition of *ρ* and $\sum_{i=1}^{n}\frac{1}{2^{i}}\leq1$, one can prove the following functional inequality:

7 $$ \begin{aligned}[b]\rho {\biggl(} f(x)- 4^{n}f {\biggl(}\frac{x}{2^{n}} { \biggr)} {\biggr)} & = \rho {\Biggl(} \sum_{i=1}^{n} \frac{1}{2^{i}} {\biggl(}2^{3i-2}f {\biggl(} \frac{x}{2^{i-1}} { \biggr)}-2^{3i}f {\biggl(} \frac{x}{2^{i}} {\biggr)} {\biggr)} { \Biggr)} \\ & \leq \frac{1}{k^{2}}\sum_{i=1}^{n} { \biggl(}\frac{k^{3}}{2} {\biggr)}^{i}\phi {\biggl(} \frac{x}{2^{i}}, \frac{x}{2^{i}} {\biggr)} \end{aligned}$$

for all $x \in V$. Now, replacing *x* by $2^{-m}x$ in (7), we obtain

$$\begin{aligned} \rho {\biggl(} 4^{m}f {\biggl(} \frac{x}{2^{m}} {\biggr)} - 4^{n+m}f {\biggl(} \frac {x}{2^{n+m}} {\biggr)} {\biggr)} & \leq k^{2m}\rho {\biggl(} f {\biggl(} \frac{x}{2^{m}} {\biggr)} - 4^{n}f {\biggl(} \frac{x}{2^{n+m}} {\biggr)} {\biggr)} \\ & \leq k^{2m-2}\sum_{i=1}^{n} { \biggl(}\frac{k^{3}}{2} {\biggr)}^{i}\phi {\biggl(} \frac{x}{2^{i+m}}, \frac{x}{2^{i+m}} {\biggr)} \\ & \leq \frac{2^{m}}{k^{m+2}}\sum_{i=m+1}^{n+m} { \biggl(}\frac{k^{3}}{2} {\biggr)}^{i}\phi {\biggl(} \frac{x}{2^{i}}, \frac{x}{2^{i}} {\biggr)} \end{aligned}$$

for all $x \in V$, which tends to zero as $m \rightarrow\infty$ because $\frac{2}{k} \le 1$ and the series of (5) converges. Thus, the sequence $\{ 4^{n}f(\frac{x}{2^{n}})\} $ is a *ρ*-Cauchy sequence for all $x \in V$ and so it is *ρ*-convergent in $X_{\rho }$ since the space $X_{\rho}$ is *ρ*-complete. Therefore we have a mapping $B:V \rightarrow X_{\rho}$ as

$$ B(x):=\text{$\rho$-} \lim_{n\rightarrow\infty} 4^{n} f {\biggl(} \frac {x}{2^{n}} {\biggr)}, \quad \textit{i.e.},\lim_{n\rightarrow\infty} \rho { \biggl(} 4^{n} f {\biggl(}\frac {x}{2^{n}} {\biggr)} - B(x) { \biggr)}=0 $$

for all $x \in V$. So, without using the Fatou property, we can see from the $\Delta_{2}$-condition that the inequality

$$\begin{aligned} \rho\bigl(f(x)-B(x)\bigr) &\leq \frac{1}{2}\rho {\biggl(} 2f(x)-2 \cdot4^{n}f {\biggl(} \frac{x}{2^{n}} {\biggr)} {\biggr)} + \frac{1}{2}\rho {\biggl(}2\cdot 4^{n}f {\biggl(} \frac{x}{2^{n}} {\biggr)} - 2B(x) {\biggr)} \\ & \leq \frac{k}{2}\rho {\biggl(} f(x)-4^{n}f {\biggl(} \frac{x}{2^{n}} {\biggr)} {\biggr)} +\frac{k}{2}\rho {\biggl(} 4^{n}f {\biggl(} \frac{x}{2^{n}} {\biggr)} - B(x) {\biggr)} \\ & \leq \frac{1}{2k}\sum_{i=1}^{n} { \biggl(} \frac{k^{3}}{2} {\biggr)}^{i} \phi {\biggl(} \frac{x}{2^{i}}, \frac{x}{2^{i}} {\biggr)} + \frac{k}{2}\rho {\biggl(} 4^{n}f {\biggl(} \frac{x}{2^{n}} {\biggr)} - B(x) {\biggr)} \end{aligned}$$

holds for all $x \in V$ and all positive integers $n>1$. Taking $n \rightarrow\infty$, one has the estimation (6) of *f* by *B*. Setting $(x,y) := (2^{-n}x, 2^{-n}y)$ in (5), we see that

$$ \rho {\biggl(} 4^{n}f {\biggl(}\frac{x+y}{2^{n}} {\biggr)} + 4^{n}f {\biggl(} \frac {x-y}{2^{n}} {\biggr)} - 2\cdot4^{n}f {\biggl(} \frac{x}{2^{n}} {\biggr)} -2\cdot 4^{n}f {\biggl(} \frac{y}{2^{n}} {\biggr)} {\biggr)} \leq k^{2n}\phi {\biggl(} \frac{x}{2^{n}}, \frac{y}{2^{n}} {\biggr)}, $$

which tends to zero as $n\rightarrow\infty$ for all $x, y \in V$. Thus, it follows from the convexity of *ρ* that

$$\begin{gathered} \rho {\biggl(}\frac{1}{7} B(x+y)+\frac{1}{7}B(x-y)- \frac {2}{7}B(x)-\frac{2}{7}B(y) {\biggr)} \\ \quad\leq \frac{1}{7}\rho {\biggl(} B(x+y)- 4^{n}f {\biggl(} \frac {x+y}{2^{n}} {\biggr)} {\biggr)} +\frac{1}{7}\rho {\biggl(} B(x-y)- 4^{n}f {\biggl(}\frac{x-y}{2^{n}} {\biggr)} {\biggr)} \\ \qquad {} + \frac{2}{7}\rho {\biggl(} B(x)- 4^{n}f {\biggl(} \frac{x}{2^{n}} {\biggr)} {\biggr)}+ \frac{2}{7}\rho {\biggl(} B(y)- 4^{n}f {\biggl(}\frac{y}{2^{n}} {\biggr)} {\biggr)} \\ \qquad{} +\frac{1}{7}\rho {\biggl(} 4^{n}f {\biggl(} \frac{x+y}{2^{n}} {\biggr)}+4^{n}f {\biggl(}\frac{x-y}{2^{n}} { \biggr)} -2\cdot4^{n}f {\biggl(}\frac {x}{2^{n}} {\biggr)}-2 \cdot4^{n}f {\biggl(}\frac{y}{2^{n}} {\biggr)} {\biggr)} \end{gathered}$$

for all $x , y \in V$ and all positive integers $n>1$. Taking the limit as $n \rightarrow\infty$, one sees that *B* is quadratic.

To show the uniqueness of *B*, we assume that there exists a quadratic mapping $B':V \rightarrow X_{\rho}$ satisfying the approximation

$$ \rho\bigl(f(x)-B'(x)\bigr)\leq\frac{1}{2k}\sum _{i=1}^{\infty} {\biggl(}\frac {k^{3}}{2} { \biggr)}^{i}\phi {\biggl(} \frac{x}{2^{i}}, \frac{x}{2^{i}} { \biggr)} \quad (x \in V). $$

Then we see from the equality $B(2^{-n}x)=4^{-n}B(x)$ and $B'(2^{-n}x)=4^{-n}B'(x)$ that

$$\begin{aligned} \rho\bigl( B(x)- B'(x)\bigr) \leq{}& \frac{1}{2}\rho { \biggl(}2\cdot4^{n} B {\biggl(}\frac{x}{2^{n}} {\biggr)}-2 \cdot4^{n}f {\biggl(}\frac{x}{2^{n}} {\biggr)} {\biggr)} \\ & + \frac{1}{2}\rho {\biggl(} 2\cdot4^{n}f {\biggl(} \frac{x}{2^{n}} {\biggr)}- 2\cdot4^{n}B' {\biggl(} \frac{x}{2^{n}} {\biggr)} {\biggr)} \\ \leq{}& \frac{k^{2n+1}}{2}\rho {\biggl(} B {\biggl(}\frac{x}{2^{n}} { \biggr)}-f {\biggl(}\frac{x}{2^{n}} {\biggr)} {\biggr)} + \frac{k^{2n+1}}{2}\rho {\biggl(} f {\biggl(}\frac{x}{2^{n}} {\biggr)}-B' {\biggl(} \frac{x}{2^{n}} {\biggr)} {\biggr)} \\ \leq{}& \frac{k^{2n}}{2}\sum_{i=1}^{\infty} { \biggl(} \frac{k^{3}}{2} {\biggr)}^{i}\phi {\biggl(} \frac{x}{2^{n+i}}, \frac{x}{2^{n+i}} {\biggr)} \\ = {}& \frac{2^{n-1}}{k^{n}}\sum_{i=n+1}^{\infty} { \biggl(} \frac {k^{3}}{2} {\biggr)}^{i}\phi {\biggl(} \frac{x}{2^{i}}, \frac{x}{2^{i}} {\biggr)} \end{aligned}$$

for all $x \in V$ and all sufficiently large positive integers *n*. Taking $n \rightarrow\infty$, we arrive at the uniqueness of *B*. This completes the proof. □

### Corollary 4

*Suppose* *V* *is a normed space with norm* $\| \cdot\|$ *and* $X_{\rho}$ *satisfies* $\Delta_{2}$-*condition*. *For given real numbers* $\theta>0 $ *and* $p>\log_{2}\frac{k^{3}}{2}$, *if* $f:V \rightarrow X_{\rho}$ *is a mapping such that*

$$ \rho\bigl(f(x+y)+f(x-y)-2f(x)-2f(y)\bigr)\leq\theta\bigl(\| x \|^{p}+ \| y\|^{p}\bigr) $$

*for all* $x, y \in V$, *then there exists a unique quadratic mapping* $B: V \rightarrow X_{\rho}$ *such that*

$$ \rho\bigl(f(x)-B(x)\bigr) \leq\frac{k^{2}\theta}{2^{p+1}-k^{3}}\|x\|^{p} $$

*for all* $x \in V$.

Next, we provide an alternative stability theorem of Theorem 3 without using both the $\Delta_{2}$-condition and the Fatou property in modular spaces.

### Theorem 4

*Suppose that a mapping* $f : V \rightarrow X_{\rho} $ *satisfies*

8 $$ \rho\bigl(f(x+y)+f(x-y)-2f(x)-2f(y)\bigr)\leq\phi(x,y) $$

*and* $\phi:V \times V \rightarrow[0, \infty) $ *is a mapping such that*

$$ \lim_{n\rightarrow\infty} \frac{\phi(2^{n}x,2^{n}y)}{4^{n}}=0, \qquad\sum _{i=0}^{\infty}\frac{\phi(2^{i}x,2^{i}x)}{4^{i}}< \infty $$

*for all* $x, y \in V$. *Then there exists a unique quadratic mapping* $B: V \rightarrow X_{\rho}$ *such that*

9 $$ \rho {\biggl(}f(x)-\frac{1}{3}f(0)-B(x) {\biggr)} \leq \frac{1}{4}\sum_{i=0}^{\infty} \frac{\phi(2^{i}x, 2^{i}x)}{4^{i}} $$

*for all* $x \in V$.

### Proof

Taking $y =x$ in (8), one has

$$ \rho\bigl(f(2x)+f(0)-4f(x)\bigr)=\rho\bigl(\tilde{f}(2x)-4\tilde{f}(x)\bigr) \leq\phi(x,x), $$

where $\tilde{f}(x)=f(x)-\frac{f(0)}{3}$, and then we obtain from the convexity of *ρ* and $\sum_{i=0}^{n-1}\frac{1}{4^{i+1}}\leq1$

$$\begin{aligned} \rho {\biggl(}\tilde{f}(x)-\frac{\tilde{f}(2^{n}x)}{4^{n}} {\biggr)} &\leq \rho {\Biggl(} \sum_{i=0}^{n-1} {\biggl(} \frac{4\tilde{f}(2^{i}x)-\tilde {f}(2^{i+1}x)}{4^{i+1}} {\biggr)} {\Biggr)} \\ & \leq \sum^{n-1}_{i=0}\frac{\rho(4\tilde{f}(2^{i}x)-\tilde {f}(2^{i+1}x))}{4^{i+1}} \\ & \leq \frac{1}{4} \sum^{n-1}_{i=0} \frac{\phi(2^{i}x, 2^{i}x)}{4^{i}} \end{aligned}$$

for all $x \in V$ and all positive integers *n*. Then, by applying a similar argument to the proof of Theorem 2, one has a *ρ*-Cauchy sequence ${\{} \frac{\tilde{f}(2^{n}x)}{4^{n}} {\}}$ and the limit function $B: V \rightarrow X_{\rho}$ defined as

$$ \text{$\rho$-} \lim_{n \rightarrow\infty} \frac{\tilde {f}(2^{n}x)}{4^{n}}=B(x), \quad \textit{i.e.}, \lim_{n \rightarrow\infty}\rho {\biggl(}\frac{\tilde{f}(2^{n}x)}{4^{n}}-B(x) {\biggr)}=0 $$

for all $x \in V$ without using the $\Delta_{2}$-condition and the Fatou property. Furthermore, one can prove that the mapping *B* satisfies the quadratic functional equation in the same way as in the proof of Theorem 3.

Now, we prove the estimation (9) of *f* by *B* without using $\Delta_{2}$-condition and the Fatou property. By using the convexity of *ρ* and $\sum_{i=0}^{n-1}\frac {1}{4^{i+1}} +\frac{1}{4} \leq1$, we obtain the following inequality:

$$\begin{aligned} \rho {\bigl(} \tilde{f}(x)-B(x) {\bigr)} &= \rho {\Biggl(} \sum _{i=0}^{n-1} {\biggl(}\frac{4\tilde{f}(2^{i}x)-\tilde {f}(2^{i+1}x)}{4^{i+1}} {\biggr)} + \frac{\tilde{f}(2^{n}x)}{4^{n}}-\frac {B(2x)}{4} {\Biggr)} \\ & \leq \sum_{i=0}^{n-1}\frac{1}{4^{i+1}} \rho\bigl(4\tilde {f}\bigl(2^{i}x\bigr)-\tilde{f}\bigl(2^{i+1}x \bigr)\bigr)+\frac{1}{4}\rho {\biggl(} \frac{\tilde {f}(2^{n-1}2x)}{4^{n-1}}-{B(2x)} {\biggr)} \\ & \leq \frac{1}{4}\sum_{i=0}^{n-1} \frac{1}{4^{i}} \phi\bigl(2^{i}x,2^{i}x\bigr) + \frac{1}{4}\rho {\biggl(} \frac{\tilde{f}(2^{n-1}2x)}{4^{n-1}}-{B(2x)} {\biggr)} \end{aligned}$$

for all $x \in V$ and all positive integers $n>1$. Taking $n \rightarrow\infty$, we arrive at the desired conclusion. □

### Corollary 5

*Let* $\phi:V \times V \rightarrow[0, \infty) $ *be a given function such that*

$$ \lim_{n\rightarrow\infty} \frac{\phi(2^{n}x,2^{n}y)}{4^{n}}=0, \qquad \phi(2x,2x)\leq4L \phi(x,x) $$

*for all* $x, y \in X$ *and for some* $L \in(0,1)$. *If* $f:V \rightarrow X_{\rho}$ *is a mapping such that*

$$ \rho\bigl(f(x+y)+f(x-y)-2f(x)-2f(y)\bigr)\leq\phi(x,y) $$

*for all* $x, y \in V$, *then there exists a unique quadratic mapping* $B: V \rightarrow X_{\rho}$ *such that*

$$ \rho {\biggl(}f(x)-\frac{1}{3}f(0)-B(x) {\biggr)} \leq\frac{1}{4(1-L)} \phi (x, x) $$

*for all* $x \in V$.

### Remark 3

In [26], the authors have shown that if the convex modular *ρ* is lower semicontinuous and $\phi:V \times V \rightarrow[0, \infty), f:V \rightarrow X_{\rho} $ with $f(0)=0$ are given functions such that

$$\begin{gathered} \rho\bigl(4f(x+y)+4f(x-y)-8f(x)-8f(y)\bigr)\leq\phi(x,y), \\ \lim_{n\rightarrow\infty} \frac{\phi(2^{n}x,2^{n}y)}{4^{n}}=0 \quad\text{and} \quad \phi(2x,2x)\leq4L \phi(x,x) \end{gathered}$$

for all $x, y \in V$ and for some $L \in(0,\frac{1}{2})$, then there exists a unique quadratic mapping $B: V \rightarrow X_{\rho}$ such that

$$ \rho\bigl(f(x)-B(x)\bigr) \leq\frac{1}{16(1-L)}\phi(x, x) $$

for all $x \in V$. In Corollary 5, we remark that since $\phi(2^{i}x, 2^{i}x) \leq(4L)^{i}\phi(x,x)$, $x \in V$, the series $\sum_{i=0}^{\infty}\frac{\phi(2^{i}x,2^{i}x)}{4^{i}}$ converges for all $x \in V$. Thus, we see that Corollary 5 is a refined stability theorem of the result above.

### Corollary 6

*Suppose* *V* *is a normed space with norm* $\| \cdot\|$. *For given real numbers* $\theta, \varepsilon>0$ *and* $p \in(-\infty, 2)$, *if* $f:V \rightarrow X_{\rho}$ *is a mapping such that*

$$ \rho\bigl(f(x+y)+f(x-y)-2f(x)-2f(y)\bigr)\leq\theta\bigl(\| x \|^{p}+ \| y\| ^{p}\bigr)+\varepsilon $$

*for all* $x, y \in V$, *then there exists a unique quadratic mapping* $B: V \rightarrow X_{\rho}$ *such that*

$$ \rho {\biggl(}f(x)-\frac{1}{3}f(0)-B(x) {\biggr)} \leq\frac{2\theta }{4-2^{p}} \|x\|^{p} +\frac{\varepsilon}{3} $$

*for all* $x \in V$, *where* $x \neq0$ *if* $p<0$.

## Conclusion

In this article, we have obtained the stability results and alternative stability results of additive functional equation and quadratic functional equation in modular spaces without using the Fatou property or the $\Delta_{2}$-condition. These generalize the results of Sadeghi [25] and Wongkum, Chaipunya and Kumam [26].
